# The Effect of Secondary Aluminum Ash on the Properties of Reactive Powder Concrete

**DOI:** 10.3390/ma16155265

**Published:** 2023-07-27

**Authors:** Wenyu Xu, Hui Wang, Xiaoning Tian

**Affiliations:** 1 Nanjing University of Science and Technology ZiJin College, Nanjing 210023, China; xuwenyu598@njust.edu.cn; 2 School of Civil Engineering and Geographic Environment, Ningbo University, Ningbo 315000, China

**Keywords:** secondary aluminum ash, rheological properties, NaCl action, thermogravimetric analysis, X-ray diffraction spectrum

## Abstract

Secondary aluminum ash is a kind of common solid waste which will pollute the environment without any treatment. In this study, the influence of secondary aluminum ash on the rheological properties and the initial setting time of fresh reactive powder concrete (RPC) are researched. Meanwhile, the mechanical properties and the drying shrinkage rates of RPC with the secondary aluminum ash are determined. The electrical parameters of RPC with the secondary aluminum ash are measured. Scanning electron microscopy is obtained to reflect the internal structure of RPC. Results show that the addition of secondary aluminum ash can lead to decreasing the fluidity and increase the yield shear stress of fresh RPC paste by varying rates of 16.1% and 58.3%, respectively. The addition of secondary aluminum ash can decrease the flexural and compressive strengths of RPC cured for 1 day by the decreasing rates of 0~18.7% and 0~19.3%. When the curing age is 28 days, the flexural and compressive strengths of RPC are increased by 0~9.1% and 0~19.1% with adding the secondary aluminum ash. The secondary aluminum ash can promote the condensation of RPC. The addition of the secondary aluminum ash can decrease the electrical resistance of RPC by an order of magnitude. The relationship between the electrical resistance and the electrical reactance fits the quadratic function equation. The electrical resistance of the pore solution increases in the form of a quadratic function with the mass ratio of the secondary aluminum ash. The dry shrinkage rates of RPC cured for 1 day and 28 days are decreased by 0~36.4% and 0~41.3% with the increasing dosages of secondary aluminum ash. As obtained from the microscopic testing results, the secondary aluminum ash can improve the compactness of hydration products.

## 1. Introduction

The primary aluminum ash is directly generated during the electrolytic aluminum or casting process [1,2]. Due to the high availability of aluminum resources (metal aluminum and aluminum oxide mass fraction) in primary aluminum ash ranging from 70% to 80%, a large amount of metal resources will be wasted [3,4]. The primary aluminum ash with a metal aluminum content exceeding 50% can not be cleaned up directly, attributed to the waste of metal resources [5,6]. Therefore, the secondary disposition should be provided to the aluminum ash.

The main method used for disposing of the primary aluminum ash is the magnetic separation method, which can separate parts containing high iron minerals from primary aluminum ash through the magnetic separation process. The disposed primary aluminum ash can be further recovered and utilized [7,8]. Moreover, plasma technology has been applied in treating the primary aluminum ash. The magnetic separation method shows the advantage of the extra high flame temperature in the plasma furnace. The acid leaching method has been applied in the processing of secondary aluminum ash [9,10]. Furthermore, the fused salt electrolysis can be used for the disposition of secondary aluminum ash. Although many methods can be used to extract metal aluminum, the cost of processing is high, and many oxides have not been used timely. In the world, about 33 million tons of secondary aluminum ash are produced per year [11,12]. Hence, the resource utilization of secondary aluminum ash is very important.

Solid waste aluminum ash can be applied in multiple fields. Vani et al. have reported that solid waste aluminum ash can be used for hydrogen production [13,14]. Lucía Grande et al. have found that solid waste aluminum ash can be applied in the extraction of alumina [15,16]. Azzeddine et al. have confirmed that solid waste aluminum ash can improve the productivity of solar distillers [17]. Although solid waste aluminum ash has been applied in several fields, as the largest consumer of materials, the construction industry should fully develop the consumption of solid waste aluminum ash.

Secondary aluminum ash (SAA) has been proven to increase the mechanical strengths and the resistance to freeze–thaw cycles and the chloride ion impermeability [18]. The addition of secondary aluminum ash can reduce the early hydration of cement, thus decreasing the internal cracks in cement-based materials [19,20]. Gireesh points out that the secondary aluminum ash can increase the mechanical strength of cement concrete with a curing age of higher than 14 days [21]. The freeze–thaw’s resistance and the chloride ion impermeability have been increased by mixing the secondary aluminum ash. Several research achievements about the cement matrix with secondary aluminum ash are reported [22,23]. Little attention has been paid to the application of secondary aluminum ash in high-performance concrete.

Reactive powder cement concrete (RPC) is manufactured by mixing a large amount of mineral admixtures. RPC shows excellent mechanical strength and durability [24,25]. The addition of waste fly ash, rice husk ash and fly ash have been used for preparing the RPC. These mineral admixtures have been reported to improve the mechanical strength and the resistance to freeze–thaw cycles [26,27]. The secondary aluminum ash may be beneficial for the performance of RPC. Simultaneously, the use of secondary aluminum ash can consume a large amount of secondary aluminum ash waste [28]. The strength and rheological properties of RPC with SAA are meaningful for the production of sustainable materials and structures. The research in this area is currently quite innovative. However, few journals about this research have been reported. 

In this study, the slump flow and the plastic viscosity of fresh RPC with different dosages of secondary aluminum ash are measured. The mechanical strengths and the drying shrinkage rates of secondary aluminum ash RPC cured for 1 day, 3 days, 7 days, 14 days and 28 days are tested. The electrical resistance of RPC with different curing ages is determined. Scanning electron microscopy, thermogravimetric analysis, and X-ray diffraction curves are acquired. This research will provide new ideas for treating secondary aluminum ash solid waste. At the same time, a new type of RPC material will be prepared by adding the secondary aluminum ash (SAA).

## 2. Materials and Methods

### 2.1. Raw Materials

Ordinary Portland cement (OPC) offered by Henan Fengbo Tianrui Cement Co., Ltd., Zhengzhou, China, is used in this study. The initial and final setting times of OPC are 102.3 min and 326.4 min. Fly ash (FA) with a density of 2.6 g/cm^3^ and the bulk density of 200 kg/m^3^, a specific surface area of 15~27 m^2^/g and an average particle size of 0.1~0.15 μm purchased from Shandong Boken Silicon Materials Co., Ltd., Zibo, China is used as a mineral admixture. Secondary aluminum ash (SAA) with an Al_2_O_3_ content ranging from 20% to 60% and SiO_2_ lower than 8% is provided from Zhengzhou Yaojuxiang Industry and Trade Co., Ltd., Zhengzhou, China. Level S95 blast furnace slag powder (BFS) with a density of 2.88 g/cm^3^, activity index above 95%, a specific surface area of 437.1 m^2^/g and a loss on ignition of 2.21% manufactured by Hebei Chuangtian Engineering Materials Co., Ltd., Shijiazhuang, China, is used as another mineral admixture. The aggregate in this study is quartz sand (produced by Fengyang Dongsheng quartz sand Co., Ltd., Fengyang, China) with particle sizes of 3.31~1.63 mm, 0.83~0.34 mm, 0.33~0.22 mm and mass ratio of 1:1.5:1. The content of SiO_2_ in quartz sand is higher than 99.5% and the apparent density of quartz sand is 2.66 g/cm^3^. The particle size and compositions of the cementitious materials are shown in Table 1 and Table 2. The flowability of fresh RPC is adjusted by polycarboxylate superplasticizer, whose water-reducing rate is 37.8%. 

### 2.2. The Manufacturing Process of Specimens

**Figure 1:**
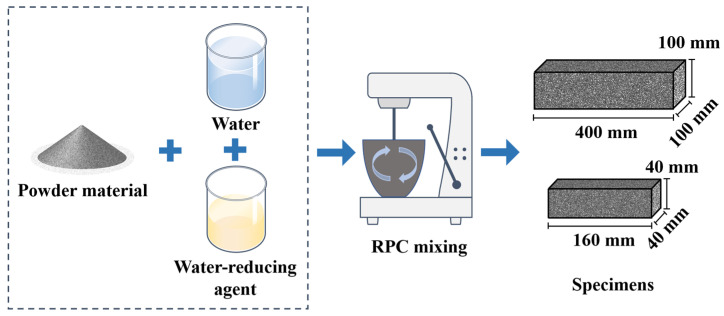
The manufacturing process of RPC specimens.

UJZ-15 mixer(produced by Shijiazhuang City Road Hang Technology Co., Ltd. Shijiazhuang, China) is used for stirring the RPC. The dry materials are added to the mixer and stirred with the mixing speed of 60 ± 2 r/min for 2 min, and then the solution is mixed with water and water reducing agent is added to the mixer, and 80 ± 2 r/min’s mixing speed is provided for the manufacturing the RPC. Specimens with sizes of 40 × 40 × 160 mm^3^ and 50 × 50 × 50 mm^3^ are prepared. The specimens are cured in the standard curing environment ((20 ± 2) °C and relative humidity of 96.2%). The manufacturing process of the RPC samples is shown in Figure 1. Table 3 shows the mixing proportions of RPC.

### 2.3. Measurement of Rheological Properties and Setting Time

**Figure 2:**
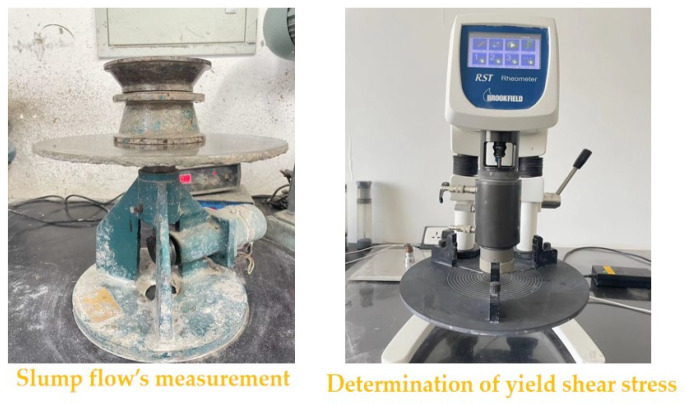
The measuring process of RPC’s rheological parameters.

The NLD-3CSA mortar dry material fluidity tester offered by Hebei Zhongxin Yida Testing Instrument Co., Ltd., Cangzhou, China, is used for the measurement of the slump flow of fresh RPC. The measuring details are described in Ma’s research [29]. The yield shear stress of fresh RPC is tested by Huck rotational rheometer provided by Shanghai Diguan Industrial Co., Ltd., Shanghai, China, with the rotational speed ranging from 0 r/min to 30 r/min. The measuring process of yield shear stress can be observed in Wang’s research [30]. The ZKS-100A mortar setting time tester provided by Shanghai Leiyun Testing Instrument Manufacturing Co., Ltd., Shanghai, China, is applied in the measurement of the initial setting time of RPC. The measuring process can be found in the Chinese standard JGJ70-90 [31]. The measuring equipment of the rheological parameters is shown in Figure 2.

### 2.4. The Determination of Mechanical Strengths

**Figure 3:**
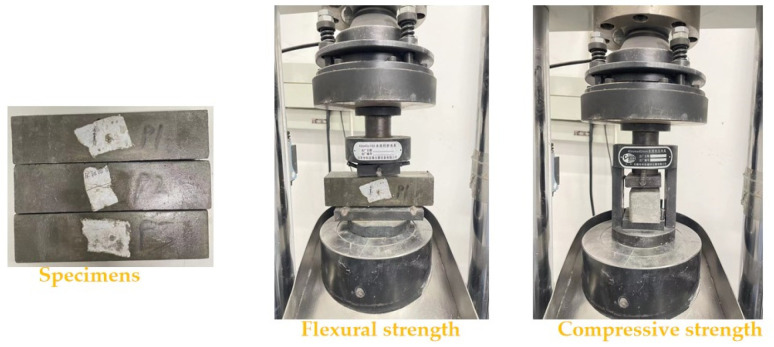
The measurement of RPC’s mechanical strength.

Specimens with the size of 40 × 40 × 160 mm^3^ are used for the determination of flexural and compressive strengths. The YAW-300C is a fully automatic bending and compression testing machine used for measuring mechanical strengths. The testing speeds of flexural and compressive strengths are 2.4 kN/s and 0.1 kN/s, respectively. The measuring process can be referred to the Chinese standard GB/T17671-1999 and Zhu’s research [32,33]. The testing process of mechanical strengths is shown in Figure 3.

### 2.5. The Determination of Electrical Parameters and Drying Shrinkage Rate

**Figure 4:**
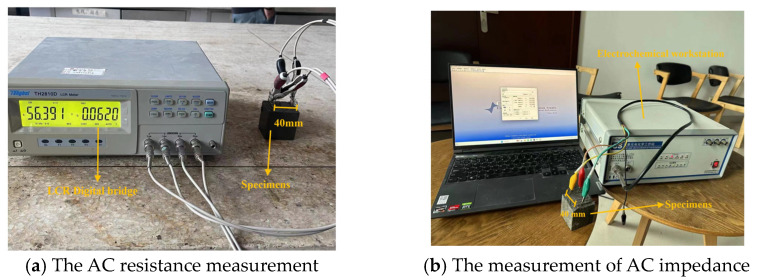
The equipment of AC electrical parameters.

Specimens with sizes of 50 × 50 × 50 mm^3^ are used for the determination of electrical parameters. The TH2830 digital bridge is used to measure the AC electrical resistance with the AC frequency of 10^5^ Hz and the voltage of 1 V. Shanghai Chenhua CHI600E electrochemical analyzer electrochemical workstation is used for the measurement of the AC impedance spectrum. The testing frequency ranges from 10^5^ Hz to 1 Hz. The AC voltage of the AC impedance spectrum is −10~10 mV. The two-electrode method is considered for the measurement of the electrical parameters. The space between the two electrical electrodes is 40 mm. The measuring process of electrical parameters is shown in Figure 4. 

The drying shrinkage rate (DSR) can be tested by the following steps. The specimens are installed on the bracket of the multimeter. The micrometer provided by Shenzhen Lide Xinmao Technology Co., Ltd., Shenzhen, China, is used for the testing of dry shrinkage values. The DSR can be obtained by Equation (1).

(1) $$DSR=\frac{L_{1}-L_{t}}{L_{1}}$$

**Figure 5:**
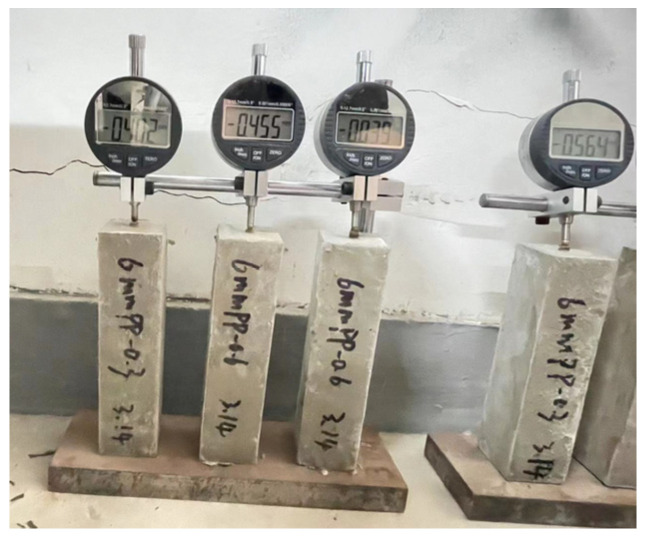
The testing process of DSR.

In Equation (1), *L*_1_ represents the initial length of the specimen, and *L_t_* means the length of the specimen at different curing ages. By this method, the *DSR* is obtained. The measurement of *DSR* is shown in Figure 5. 

### 2.6. The Scanning Electron Microscopy and XRD Experiments

The samples are removed from the inner parts of the specimen. The samples with the maximum diameter of 3 mm and the minimum diameter of 0.5 mm are used for observing the scanning electron microscopy photos. All samples are dried in the Li Chen vacuum drying chamber provided by China Experimental Instrument Sales Center, Beijing, China. The samples are moved to the vacuum spraying chamber for spraying gold. After that, the Zeiss scanning electron microscope is applied to observe the SEM photos of samples. The residual samples are pulverized to powder. The powdered samples are used for obtaining the X-ray diffraction spectrums by the TD-3500 X-ray diffractometer purchased from Wuxi Lingen Electromechanical Equipment Co., Ltd., Wuxi, China.

## 3. Results and Discussions

In the experimental results of this paper, the percentage of SAA means the percentage of (the total mass of fly ash and SAA). 

### 3.1. The Rheological Properties of Fresh RPC

**Figure 6:**
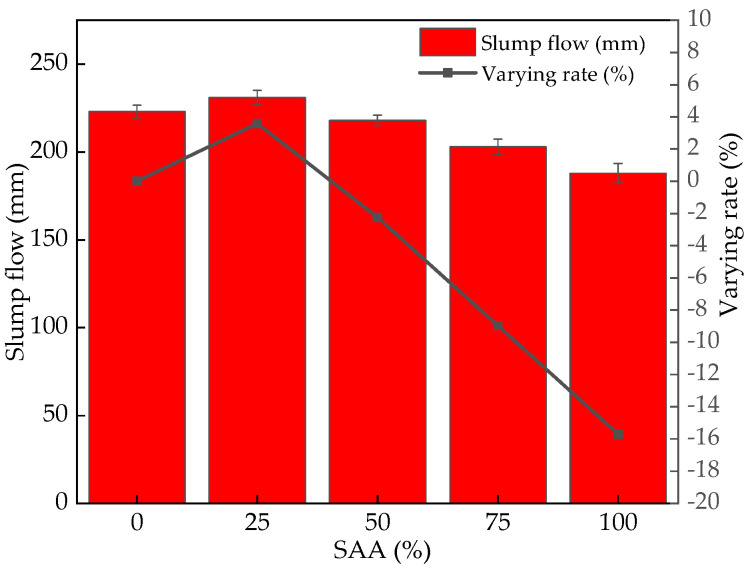
The slump flow and the corresponding increasing rate of fresh RPC with SAA.

The slump flow of fresh RPC mixing with different content of SAA is illustrated in Figure 6. As observed from Figure 6, the slump flow increases with the addition of SAA ranging from 0% to 25%, which is attributed to the lower specific surface area than the FA [34,35]. FA in RPC can absorb higher specific surface area, leading eventually to decreasing the slump flow of fresh RPC [36]. While the increasing dosage of SAA with a mass ratio of 25~100% can decrease the slump flow of fresh RPC due to the ball effect of SAA [37,38]. The growth rate of the slump flow firstly increases from 0% to 3.59% and then decreases from 3.59% to −15.7%.

**Figure 7:**
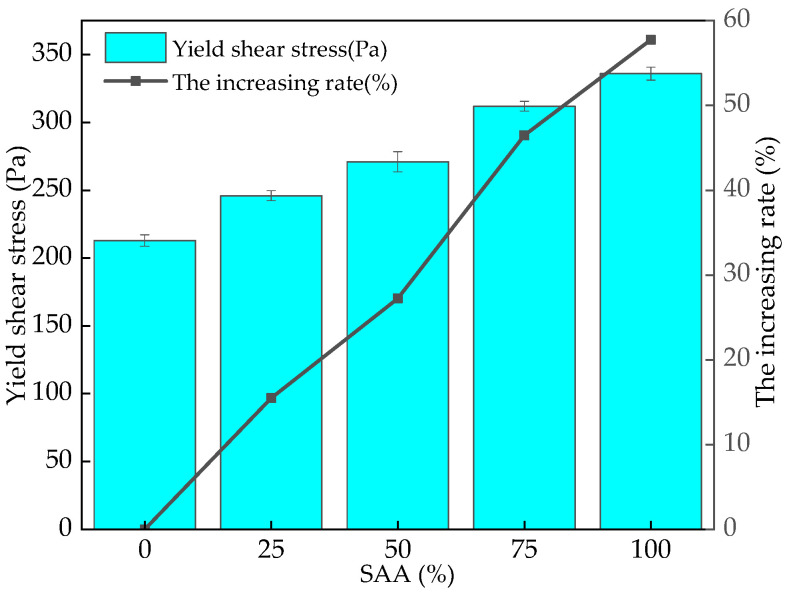
The yield shear stress and the corresponding increasing rate of fresh RPC with SAA.

The yield shear stress of fresh RPC mixing with different content of SAA is shown in Figure 7. It can be obtained from Figure 7 that the yield shear stress of fresh RPC continues to increase with the increasing dosages of SAA. The increasing rate of the yield shear stress of fresh RPC increases from 0% to 57.75%. This is ascribed to the fact that the SAA with a high specific surface area can absorb more free water, and then a large number of flocculent substances form in the fresh RPC, leading to an increase in yield shear stress [33,39].

### 3.2. The Initial Setting Time of RPC

**Figure 8:**
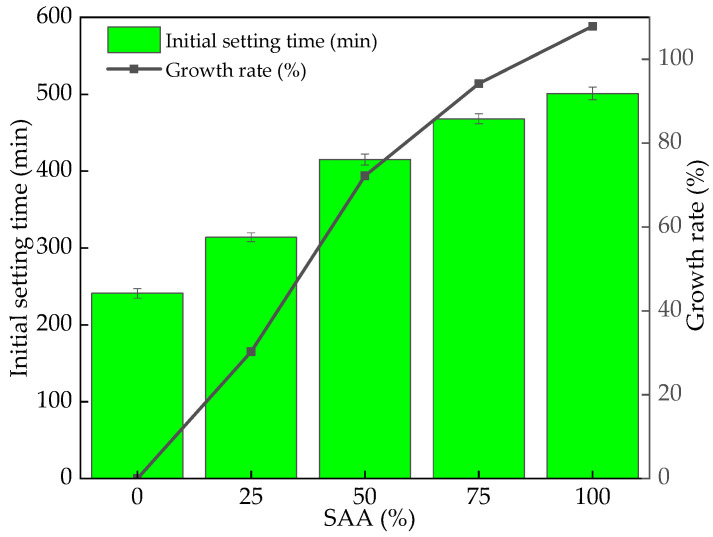
The initial setting time of fresh RPC with SAA.

The initial setting time of fresh RPC mixing with different content of SAA is shown in Figure 8. It can be found in Figure 8 the initial setting time of fresh RPC increases with the increasing dosage of SAA. This is ascribed to the fact that SAA can delay the hydration of the paste by reducing the volcanic ash reaction with excess Ca(OH)_2_ generated during the cement hydration process, thereby extending the setting time [40,41]. The initial setting time can be increased with the maximum increasing rate of 112.3%.

### 3.3. The Mechanical Strengths of RPC

**Figure 9:**
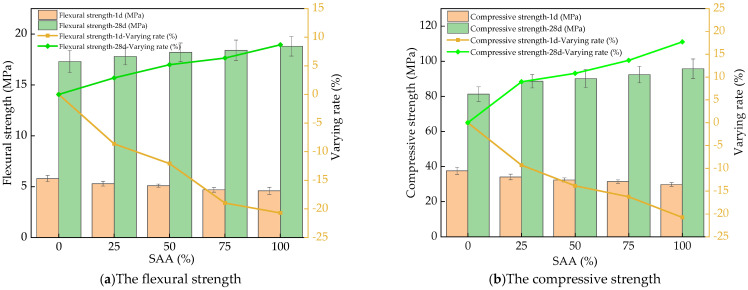
The mechanical strengths of RPC with SAA.

The flexural and compressive strengths of RPC mixing with different content of SAA are exhibited in Figure 9. When the curing age is 1 day, the flexural and compressive strengths of RPC decrease with the increasing content of SAA. When the curing age of RPC is 28 days, the mechanical strengths of RPC are increased by the addition of SAA. This is attributed to the fact that, when the curing age is l day, the added SAA can make ettringite in calcium monosulfoaluminate form in cement with hexagonal crystal [42]. Therefore, the mechanical strengths of RPC cured for 1 day decrease with the increasing dosage of SAA with the decreasing rates of 0~18.7% and 0~19.3%. Meanwhile, when the curing age reaches 28 days, the flexural and compressive strengths are increased by adding the SAA with increasing rates of 0~9.1% and 0~19.1%. The Al_2_O_3_ of SAA can react with the primary hydration products (Ca(OH)_2_) in cement, forming calcium aluminates which can accelerate the secondary hydration of cement can promoting the corresponding hydration degree. Therefore, the mechanical strengths of RPC are increased by adding SAA [8].

### 3.4. The Electrical Parameters of RPC

**Figure 10:**
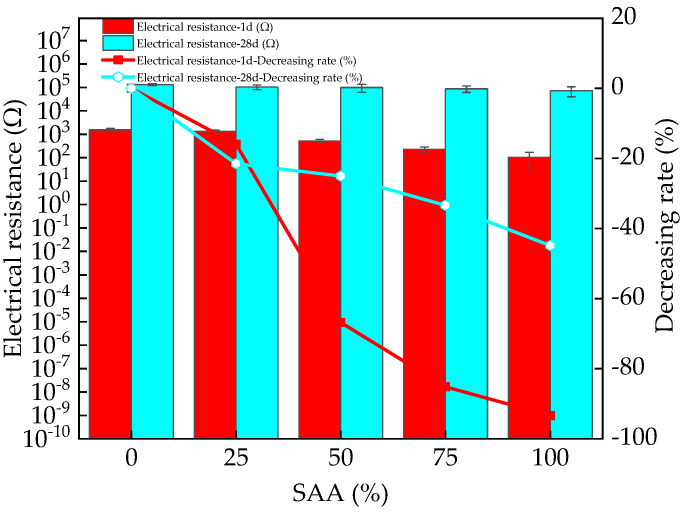
The electrical resistance of RPC with SAA.

The electrical resistance of RPC is shown in Figure 10. It can be found in Figure 10 that the electrical resistance of RPC decreases with the increasing content of SAA. This is attributed to the fact that the addition of SAA contains a certain amount of metallic aluminum, which can provide higher free electrons, thus improving the electrical conduction of RPC and decreasing the electrical resistance [43,44]. When the curing age increases from 1 day to 28 days, the electrical resistance increases with the increasing rate of 7870~70,406%. This is ascribed to the improved hydration degree by the increased curing age [45]. The increased hydration degree consumes free water leading to a decrease in the concentration of pore solution and an increase in the electrical resistance. 

**Figure 11:**
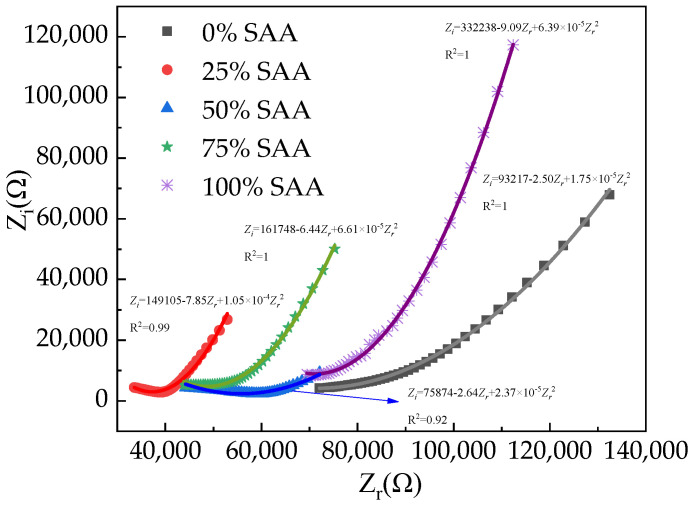
The AC electrical impedance spectrum curves of RPC with SAA.

The AC electrical impedance spectrum curves are depicted in Figure 11. In this Figure, *Z_r_* represents the AC electrical resistance, and *Z_i_* means the AC electrical reactance. This is ascribed to the fact that the cement matrix consists of multiphase (the phases of liquid, solid, and gas). The interface between different phases will generate capacitive reactance, thus forming the bulk resistance of cement-based material.

As found in Figure 11, the AC electrical reactance decreases first and then increases with the AC electrical resistance. The relationship between *Z_r_* and *Z_i_* fits the function. The fitting degrees of AC electrical impedance spectrum curves are higher than 0.92, which ensures the accuracy of the fitting function. 

**Figure 12:**
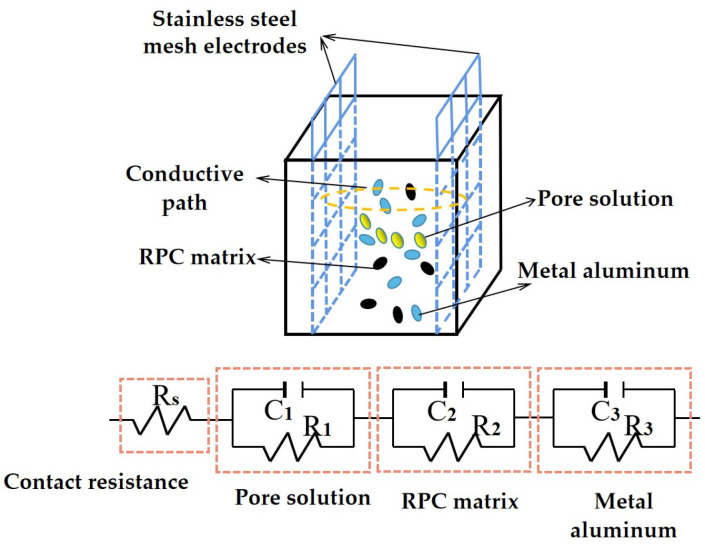
The equivalent circuit of RPC.

The AC electrical equivalent circuit of the AC electrical impedance spectrum curves is shown in Figure 12. As observed in Figure 12, the AC electrical equivalent circuit consists of four parts. The first part is the contact electrical resistance (the electrical resistance between the RPC and the electrodes). The other parts are the parallel electrical resistance and capacitance of the pore solution, the RPC matrix and the metal aluminum. The Chi of the AC electrical equivalent circuit is lower than 0.016, indicating the rationality of equivalent circuit diagrams.

**Figure 13:**
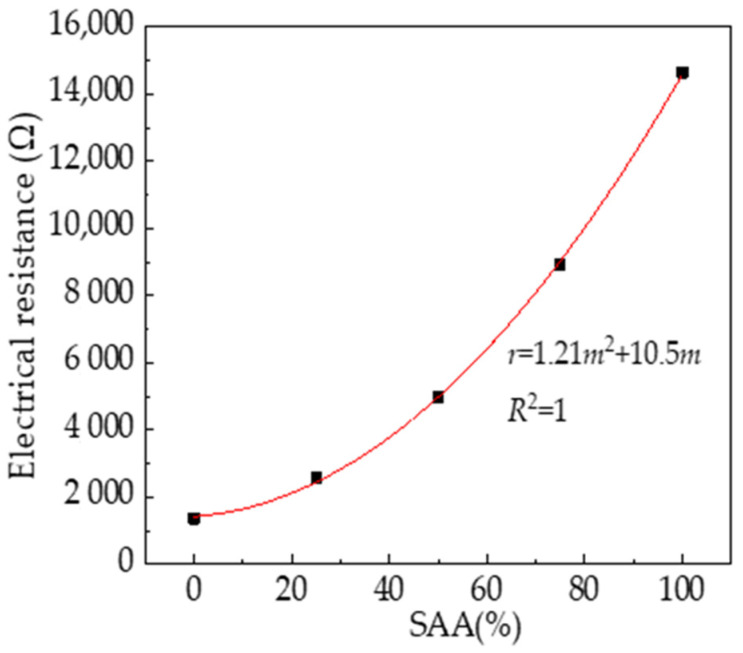
The pore solution’s electrical resistance of RPC with SAA.

The pore solution’s electrical resistance of RPC with SAA calculated by the equivalent circuit is shown in Figure 13. As shown in Figure 13, the electrical resistance of the pore solution increases in the form of a quadratic function with the mass ratio of SAA. This is due to the fact that the addition of SAA can promote the hydration degree of cement, thus decreasing the electrical conduction of the pore solution. Therefore, the electrical resistance increases with the increasing dosages of SAA. This indirectly reflects that SAA can reduce the porosity of RPC and achieve the effect of increasing RPC’s mechanical strengths.

### 3.5. The DSR of RPC

**Figure 14:**
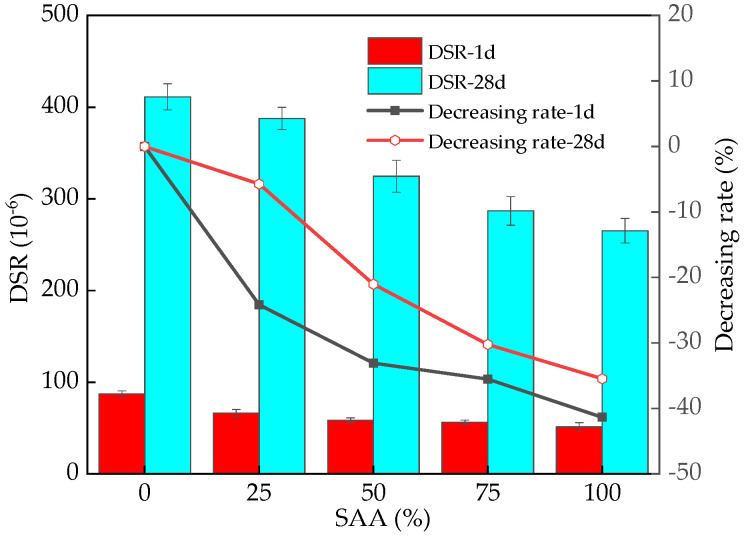
The DSR of RPC with SAA.

The DSR of RPC is shown in Figure 14. As illustrated in Figure 14, the DSR of RPC decreases with the increasing dosage of SAA due to the fact that the addition of SAA can delay the setting and early hydration of cement, thus reducing the loss of free water and decreasing the DSR of RPC. Moreover, the increased curing age demonstrates an increasing effect on the DSR of RPC due to the decreased free water and increased hydration products [46,47]. The hydration products lead to the blocking of the conductive channel and cause a decrease in electrical conductivity, which increases the electrical resistance of RPC. The variation rate of RPC’s DSR cured for 1 day ranges from 0% to 35.48%. When the RPC is cured for 28 days, the variation rate of RPC ranges from 0% to 41.43% by adding the SAA.

### 3.6. The SEM of RPC

**Figure 15:**
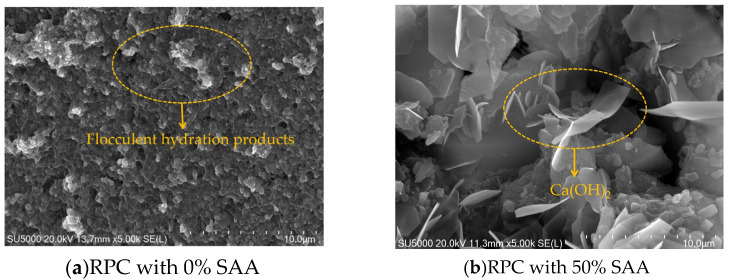
The SEM of RPC with SAA.

The SEM photos of RPC with 0% SAA and 50% SAA are shown in Figure 15. The RPC specimens are cured for 28 days. As found in Figure 15, the flocculent hydration products are discovered. The addition of SAA can significantly change the microscopic morphology of the specimen, the flocculent hydration products decrease, and large quantities of hexagonal-plate calcium hydroxide (Ca(OH)_2_) are produced. Moreover, as can be found in Figure 15, the increased hydration products can improve the compactness of RPC cured for 28 days, thus increasing the mechanical strength.

## 4. Conclusions

The fluidity of fresh RPC is decreased, and the corresponding yield shear stress is increased by adding the secondary aluminum ash with varying rates of −15.7~3.59% and 0~57.75%.

The flexural and compressive strengths of RPC cured for 1 day can be decreased by the increasing dosages of secondary aluminum ash with decreasing rates of 0~18.7% and 0~19.3%. When the RPC specimens are cured for 28 days, the flexural and compressive strengths of RPC are increased by 0~9.1% and 0~19.1% with the increasing dosages of secondary aluminum ash. The initial setting time can be increased with the maximum increasing rate of 112.3%.

The addition of secondary aluminum ash can improve the electrical conduction of RPC. Additionally, the electrical resistance of the pore solution inner RPC is increased by adding the secondary aluminum ash, leading to decreasing the pores’ volume of RPC.

The dry shrinkage rates of RPC cured for 1 day and 28 days are decreased by 0~35.48% and 0~41.3% with the increasing dosages of secondary aluminum ash.

When the curing age is 28 days, the secondary aluminum ash can improve the compactness of hydration products, thus improving the mechanical strength.

## Figures and Tables

**Table 1:** The accumulated pass rate of the binder materials (%).

Types	Particle Size/μm						
	0.3	0.6	1	4	8	64	360
OPC	0.11	0.32	2.4	15.3	28.3	93.4	100
BFS	0.042	0.13	3.28	19.38	35.17	98.18	100
FA	31.28	58.49	82.38	100	100	100	100
Quartz sand	0	0	0	0	0.039	23	100
SAA	0.05	0.21	0.59	1.13	3.93	25.9	87.22

**Table 2:** Chemical composition of the cementitious materials (%).

Types	SiO_2_	Al_2_O_3_	Fe_x_O_y_	MgO	CaO	SO_3_	K_2_O	Na_2_O	Loss on Ignition
OPC	20.63	5.56	3.77	1.79	61.60	2.78	-	-	3.08
BFS	33.73	14.78	0.49	9.72	36.60	0.29	-	-	-
SF	90.07	0.21	0.62	0.23	0.43	0.19	-	-	-
FA	39.48	28.76	4.25	0.52	3.29	-	0.36		21.11
Quartz sand	99.00	-	0.19	-	-	-	-	-	-
SAA	4.56	78.67	3.87	5.65	1.49	-	-	0.89	-

**Table 3:** The mixing proportions of RPC (kg/m^3^).

Water	OPC	SAA	FA	BFS	Quartz Sand	Water-Reducer
244.4	740.7	0	370.3	111.1	977.9	16.3
244.4	740.7	92.6	277.7	111.1	977.9	16.3
244.4	740.7	185.2	185.2	111.1	977.9	16.3
244.4	740.7	277.7	92.6	111.1	977.9	16.3
244.4	740.7	370.3	0	111.1	977.9	16.3

## Data Availability

The data used to support the findings of this study are available upon request.

## References

[1] Ozerkan N.G., Maki O.L., Anayeh M.W., Tangen S. (2014). The effect of aluminium dross on mechanical and corrosion properties of concrete. Int. J. Innov. Res. Sci. Eng. Technol..

[2] David O., Opeyemi J., Adekunle M., Babatunde F. (2019). Influence of secondary aluminum dross (SAD) on compressive strength and water absorption capacity properties of sandcrete block. Cogent. Eng..

[3] Panditharadhya B.J., Sampath V., Shankar A.U.R. (2018). Mechanical properties of pavement quality concrete with secondary aluminium dross as partial replacement for ordinary portland cement. Mater. Sci. Eng..

[4] Busari A., Joseph F., Ajayi S., Alayande T., Nwachukwu J. (2019). Index properties of aluminum dross modified pavement geo-material. J. Phys..

[5] Satish R., Neeraja D. (2016). Mechanical and durability aspects of concrete incorporating secondary aluminium slag. Resour. Effic. Technol..

[6] Ewais E.M.M., Khalil N.M., Amin M.S. (2009). Utilization of aluminum sludge and aluminum slag (dross) for the manufacture of calcium aluminate cement. Ceram. Int..

[7] Zhang Y., Guo C.H. (2018). Effects of AlN hydrolysis on fractal geometry characteristics of residue from secondary aluminium dross using response surface methodology. T. Nonferr. Metal. Soc..

[8] Arimanwa J.I., Onwuka D.O. (2012). Prediction of the Compressive Strength of Aluminum Waste–Cement Concrete Using Scheffe’s Theory. Mater. Sci..

[9] Ren Y.C., Wu F.H., Qu G.F. (2023). Extraction and preparation of metal organic frameworks from secondary aluminum ash for removal mechanism study of fluoride in wastewater. J. Mater. Res. Technol..

[10] Zhang J.J., Liu B. (2022). Theoretical and experimental on the thermodynamic, kinetic and phase evolution characteristics of secondary aluminum ash. J. Mater. Res. Technol..

[11] Xiao L.Y., Wang Y., Zheng R.F. (2023). Identification of recycling pathways for secondary aluminum dross with integrated hybrid life cycle assessment. Resour. Conserv. Recycl..

[12] Zuo Z.P., Lv H., Li R.B. (2021). A new approach to recover the valuable elements in black aluminum dross. Resour. Conserv. Recycl..

[13] Vani N.A., Nobuo H., Noriaki W. (2021). Local initiative hydrogen production by utilization of aluminum waste materials and natural acidic hot-spring water. Appl. Energy.

[14] Nuray K.A., Suha O.M. (2021). Microhydrogen production with water splitting from daily used waste aluminum. Int. J. Hydrogen Energy.

[15] Lucía G., Miguel Á.V. (2023). Synthesis strategies of alumina from aluminum saline slags. Process. Saf. Environ..

[16] Siti M.M., Nurul A.R. (2022). Comparison on the physicochemical properties of alumina extracted from various aluminum wastes. Mater. Today.

[17] Azzeddine B., Abd E.K., Mohamed A. (2023). Improving the freshwater productivity of hemispherical solar distillers using waste aluminum as store materials. J. Energy Storage.

[18] Trinet Y. (2019). The influence of aluminum dross on mechanical and corrosion properties of cement paste: PART I. Eng. Technol..

[19] Zhang Y.B., Lin K., Su Z.J. (2023). Self-driven hydrolysis mechanism of secondary aluminum dross (SAD) in the hydrometallurgical process without any additives. Chem. Eng. J..

[20] Ayobami B., Jacques S., Williams K. (2020). Data on the engineering properties of aluminum dross as a filler in asphalt. Data. Brief..

[21] Gireesh M., Sujay R., Sreedhara B.M., Manu D.S. (2016). Investigation of concrete produced using recycled aluminium dross for hot weather concreting conditions. Resour. Effic. Technol..

[22] Rotana H., Claudia P.O. (2019). On utilization and mechanisms of waste aluminium in mitigating alkali-silica reaction (ASR) in concrete. J. Clean. Prod..

[23] Socrates P.M.P., Juan M.G.C. (2023). Influence of the secondary aluminum chip on the physical and mechanical properties of concrete. Innov. Infrastruct. Solut..

[24] Kočí V., Vejmelková E., Koňáková D., Pommer V., Grzeszczyk S., Matuszek-Chmurowska A., Mordak A., Černý R. (2023). Basic physical, mechanical, thermal and hygric properties of reactive powder concrete with basalt and polypropylene fibers after high-temperature exposure. Constr. Build. Mater..

[25] Hong X., Wang H., Shi F. (2020). Influence of NaCl freeze thaw cycles and cyclic loading on the mechanical performance and permeability of sulphoaluminate cement reactive powder concrete. Coatings.

[26] Xu J., Wang H., Wang W., Shi F. (2023). The Influence of CO_2_-Cured Incinerated Waste Fly Ash on the Performance of Reactive Powder Concrete. Coatings.

[27] Cai Z., Ren J., Shen G., Jin C., Gu X., Cheng W., Wang H. (2023). The Influence of Assembly Unit of Fibers on the Mechanical and Long-Term Properties of Reactive Powder Concrete. Coatings.

[28] Mahinroosta M., Allahverdi A. (2018). Enhanced alumina recovery from secondary aluminum dross for high purity nanostructured γ-alumina powder production: Kinetic study. J. Environ. Manag..

[29] Ma Z.M., Liu M., Duan Z.H., Liang C.F., Wu H.X. (2018). Effects of active waste powder obtained from C&D waste on the microproperties and water permeability of concrete. J. Clean. Prod..

[30] Wang H., Shi F., Shen J., Zhang A., Zhang L., Huang H., Liu J., Jin K., Feng L., Tang Z. (2021). Research on the self-sensing and mechanical properties of aligned stainless steel fifiber reinforced reactive powder concrete. Cem. Concr. Compos..

[31] (2009). Standard for Test Method of Basic Properties of Construction Mortar.

[32] (1999). Method of Testing Cements-Determination of Strength.

[33] Zhu X.H., Yang J.Z., Yang Y.F. (2022). Pyrometallurgical process and multipollutant co-conversion for secondary aluminum dross: A review. J. Mater. Res. Technol..

[34] Elseknidy M., Salmiaton A. (2020). A Study on Mechanical Properties of Concrete Incorporating Aluminum Dross, Fly Ash, and Quarry Dust. Sustainability.

[35] Nduka D., Ede A. (2020). Mechanical and Water Absorption Properties of Normal Strength Concrete (NSC) Containing Secondary Aluminum Dross (SAD). Int. J. Eng. Res. Africa.

[36] Li Z.B., Li H.Q., Huang X.Z., Wu W.F. (2023). Removal of nitrides and fluorides from secondary aluminum dross by catalytic hydrolysis and its mechanism. Heliyon.

[37] Huang X.L., Mahendranath A., Robert F. (2014). Characterization of salt cake from secondary aluminum production. J. Hazard. Mater..

[38] Mostafa M., Ali A. (2018). A promising green process for synthesis of high purity activated-alumina nanopowder from secondary aluminum dross. J. Clean. Prod..

[39] Li T.X., Mohamed H., Kazunori K., Kozo S. (2006). Performance of secondary aluminum melting: Thermodynamic analysis and plant-site experiments. Energy.

[40] Yuan L., Qu G.F. (2022). Reuse of secondary aluminum ash: Study on removal of fluoride from industrial wastewater by mesoporous alumina modified with citric acid. Environ. Technol. Innov..

[41] Mostafa M., Ali A. (2018). Hazardous aluminum dross characterization and recycling strategies: A critical review. J. Environ. Manag..

[42] Panditharadhya B.J., Mulangi R.H., Ravi Shankar A.U. (2023). Mechanical properties of pavement quality concrete with aluminium industry waste as a binder. Mater. Today.

[43] Javali S., Chandrashekar A.R., Naganna S.R., Manu D.S. (2017). Eco-concrete for sustainability: Utilizing aluminum dross and iron slag as partial replacement materials. Clean. Technol. Environ. Policy.

[44] Tsakiridis P.E., Oustadakis P., Agatzini-Leonardou S. (2014). Black dross leached residue: An alternative raw material for Portland cement clinker. Waste Biomass Valorization.

[45] Haneef S.M., Harish P. (2016). An Experimental Investigation on Use of Secondary Aluminium Dross in Cement Concrete. Eng. Technol..

[46] Pereira D.A., De A.B., Castro F. (2000). Mechanical behaviour of Portland cement mortars with incorporation of Al-containing salt slags. Cem. Concr. Res..

[47] Noori A.N., Jassim A.M., Heba S.K., Arshid N. (2021). Investigation of lightweight structural materials produced using aluminum scraps with cement mortar. J. Appl. Eng. Sci..

